# Employment of GIS techniques to assess the long-term impact of tillage on the soil organic carbon of agricultural fields under hyper-arid conditions

**DOI:** 10.1371/journal.pone.0212521

**Published:** 2019-02-19

**Authors:** ElKamil Tola, Khalid A. Al-Gaadi, Rangaswamy Madugundu

**Affiliations:** 1 Precision Agriculture Research Chair, King Saud University, Riyadh, Saudi Arabia; 2 Department of Agricultural Engineering, College of Food and Agriculture Sciences, King Saud University, Riyadh, Saudi Arabia; Potsdam Institute for Climate Impact Research, GERMANY

## Abstract

A study on six 50 ha agricultural fields was conducted to investigate the effect of conservation tillage practices on the long-term (1990–2016) changes in the soil organic carbon (SOC) content of the topsoil layers (0–10 cm) of agricultural fields. The experimental fields were selected from the 49 fields of the Tawdeehiya Arable Farm (TAF), located 200 kilometers southeast of Riyadh, the capital city of the Kingdom of Saudi Arabia. Data sets from laboratory determined SOC and the corresponding Landsat images generated vegetation indices, namely, the Normalized Difference Vegetation Index (NDVI) and the Bare Soil Index (BSI), were utilized for the prediction of SOC using multivariate regression techniques. Long-term changes in the SOC content of the experimental fields, as a result of different tillage practices, were also studied. The developed SOC prediction models exhibited high accuracy indicated by R^2^ values ranging from 0.73 to 0.85, RMSE values of 0.34 to 0.85 g kg^-1^ and P-values of less than 0.0001. The cross-validation results (R^2^ of 0.61–0.70, RMSE value of 0.34–0.85 g kg^-1^ and P-values of less than 0.0001) confirmed the high accuracy of the developed SOC prediction models. Results also revealed that the change in the SOC content was clearly associated with soil tillage practices. On the average, 76% of the all agricultural fields in the experimental farm showed a decrease of up to 24 g kg^-1^ in their SOC content after 10 years (1990–2000) of continuous conventional tillage practices. On the other hand, an average increase of up to 37 g kg^-1^ in the SOC content was observed in 88% of the studied fields at the end of the study period (2016), where conservation tillage was a continous and consistent practice in the experimental farm.

## Introduction

Soil organic matter (SOM) plays an important role in the stabilization of soil structure, retention and release of plant nutrients, infiltration and storage of water in the soil; therefore, it is an essential element for soil health and fertility and food production [1]. The soil organic carbon (SOC) refers to the carbon fraction of the SOM [2]. As a source of energy and nutrients for microorganisms in the soil, the SOC is one of the most important soil constituents that have a significant impact on plant growth and development, and is considered as a key indicator of soil quality and fertility [3]. Monitoring and improving the SOM/SOC content in the soil are essential practices in maintaining the sustainability of agricultural ecosystems and quality of the environment [4]. Studies showed that permitting low concentrations of organic matter in the soil could cause degradation in the soil productive capacity of agricultural crops due to the degradation of soil physical properties and nutrient cycling mechanisms [5]. Dikgwatlhe et al. [6] reported that soil degradation and associated decline in SOC have become a major concern in conventional farming systems due to the significant reduction in crop yields.

Advances in remote sensing (RS), geographical information systems (GIS) technologies and analytical software programs provide fast, non-destructive and cost-effective methods for estimating the concentrations of organic carbon in agricultural soils [7, 8]. Examples of these analytical software programs are the Near-infrared Spectral Analysis Software–NSAS [9] and the Multiskan MS and Genesis Lite software [10]. Digital soil mapping and regression analysis provide efficient tools, which can use only a limited amount of SOC data, to predict SOC at a large-scale level. Different multivariate techniques, such as Principal Component Analysis (PCA), Multivariate Analysis of Variance (MANOVA), and Multidimensional scaling, have been successfully utilized for the prediction of SOC using soil reflectance data extracted from spectroscopy and satellite images [11, 12, 13, 14].

Frequent tillage causes deterioration of soil structure, which can affect the stability and formation of soil aggregates by increasing soil disturbance [15]. Excessive soil disturbance can significantly accelerate the mineralization of SOM, which in turn, can increase carbon dioxide release rate. In addition to increasing the concentration of the atmospheric greenhouse gases, increasing carbon dioxide emissions from agricultural soils, due to the increased soil disturbance, can introduce a negative influence on agriculture, productivity of natural resources and sustainability [16]. Reda [17] concluded that intensive/conventional tillage practices could induce significant losses of soil C in the form of CO_2_ by breaking soil aggregates, exposing organic matters to the microbes, incorporation and mixing of crop residues and improving aeration. Therefore, the adoption of conservation tillage practices, associated with the maintenance of crop residues on the soil surface, can greatly contribute into the improvement of soil carbon levels. In general, conservation tillage practices have been defined as any plowing systems that reduce the loss of soil and water compared to conventional tillage, and they include reduced tillage, minimum tillage and zero tillage systems. The adoption of conservation tillage can lead to significant increases in the carbon content of agricultural soils, reversing the decline resulting from the long-term application of conventional tillage practices [18].

Literature indicated that conservation tillage, which improves soil water storage and increases SOM content [19, 20], was most beneficial when practiced in arid and semi-arid areas, where soil moisture and SOM are considered as key factors to consider [21, 22]. Also, Zarea [23] reported that conservation tillage has a considerable potential for stabilizing production in semi-arid zones. Therefore, the main goal of this study was to assess the long-term impact of conservation tillage on the SOC content of agricultural fields under hyper-arid climatic conditions of Saudi Arabia, where a very little research work has been done in this regard. The specific objectives of the study were: (1) to employ multivariate regression techniques for the prediction of SOC using vegetation indices extracted from Landsat images, and (2) to investigate the impact of conventional and conservation tillage practices on the temporal dynamics of SOC content of the selected agricultural fields for a period of 26 years (1990–2016).

## Materials and methods

### Study area

**Fig 1:**
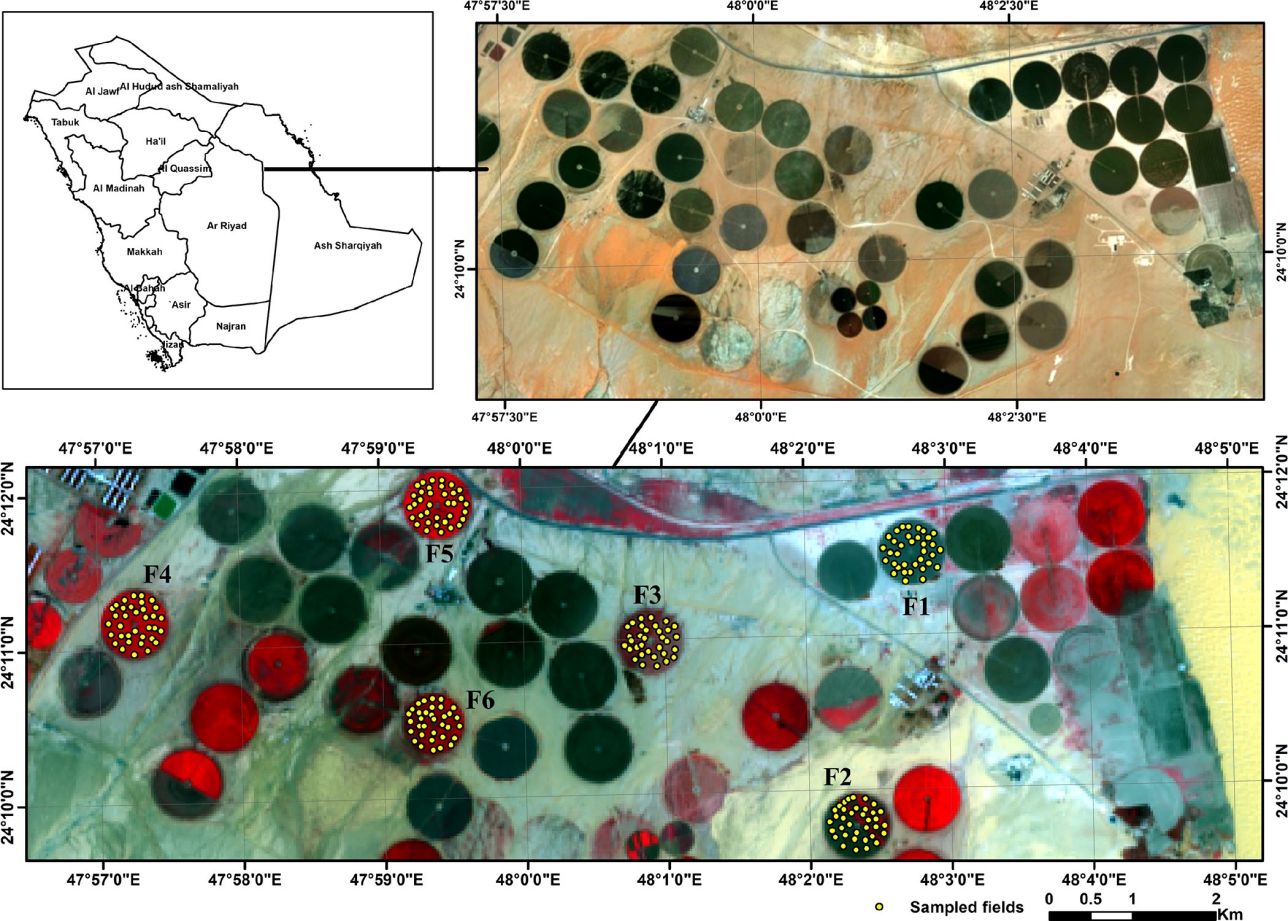
Location map of the experimental fields overlaid by the sampling points.

The field study was conducted in the Tawdeehiya Arable Farm (TAF) located 200 km southeast of Riyadh, the capital city of Saudi Arabia, between the the latitudes of 24° 10′ 22.77″ and 24° 12′ 37.25″ N and the longitudes of 47° 56′ 14.60″ and 48° 05′ 08.56″ E (Fig 1). The collection of field data was performed under permission of Mr. Alan King, the Farm Manager of Tawdeehiya Arable Farm. The TAF farm was located in a region of hyper-arid climate with a mean maximum temperature of 40 ± 2°C during summers, a mean minimum temperature of 15 ± 3°C during winters and a mean air temperature of 35°C. The study farm fell in a very low precipitation zone of 90 mm mean annual rainfall occurred in the period from November to February. The soil of the study area was characterized as mainly sandy loam with electrical conductivity (EC) values ranging from 1.85 to 2.57 mS cm^-1^ and pH values between 7.8 and 8.1 (Table 1). EC values of the farm irrigation water ranged from 2.1 to 5.0 mS cm^-1^ and pH values of 7.5–7.7. The major crops cultivated in the experimental farm were wheat, barley, alfalfa, Rhodes grass and corn. The alfalfa was cultivated as a biannual crop (November to October), with an average of 30–35 days for each harvest during summer periods and 45–60 days during winter periods. The Rhodes grass was grown as an annual crop (February to December), with an average of 28–35 days for each harvest during summer periods and 55–70 days during winter periods. However, wheat and barley crops were cultivated as seasonal winter crops during the period from November to February/March. Out of the 47 agricultural fields (≈ 50 ha each) of the experiemental farm, six fields (F1 to F6) were randomly selected and considered for this study (Fig 1).

**Table 1:** Description of the experimental fields.

Field Number	Field ID	Area (ha)	Soil type	Soil		Irrigation Water	
				EC (mS cm^-1^)	pH	EC (mS cm^-1^)	pH
F1	TE-2	50	Sandy Loam	1.90	8.08	2.13	7.65
F2	P4-5	50	Sandy Loam	2.33	7.79	2.52	7.61
F3	P12A	50	Sandy Loam	1.85	7.90	4.05	7.54
F4	P8	50	Sandy Loam	1.85	7.88	4.84	7.39
F5	P2	50	Sandy Loam	2.09	7.91	3.45	7.52
F6	P14	50	Sandy Loam	2.57	7.80	5.01	7.45

### Tillage practices

The experimental farm was under a continuous practice of conventional tillage systems until the late 90’s of the last century, when conservation tillage practices were introduced for the first time on the farm. Based on the cultivated crops, the major soil tillage implements used during the conventional tillage period were the John Deere 650 Disk, CASE IH 5150 and GREGOIRE BESSON disk harrow and HORSCH TERRANO FG cultivator. On the other hand, conservation tillage practices mainly included direct seeding of forages (alfalfa, barley and Rhodes grass) and wheat crop using the VÄDERSTAD RAPID F 800 DIRECT SEED DRILL. Sowing of corn crop, however, was achieved using MONOSEM 8-row No-Till planter.

### Experimental procedure

**Fig 2:**
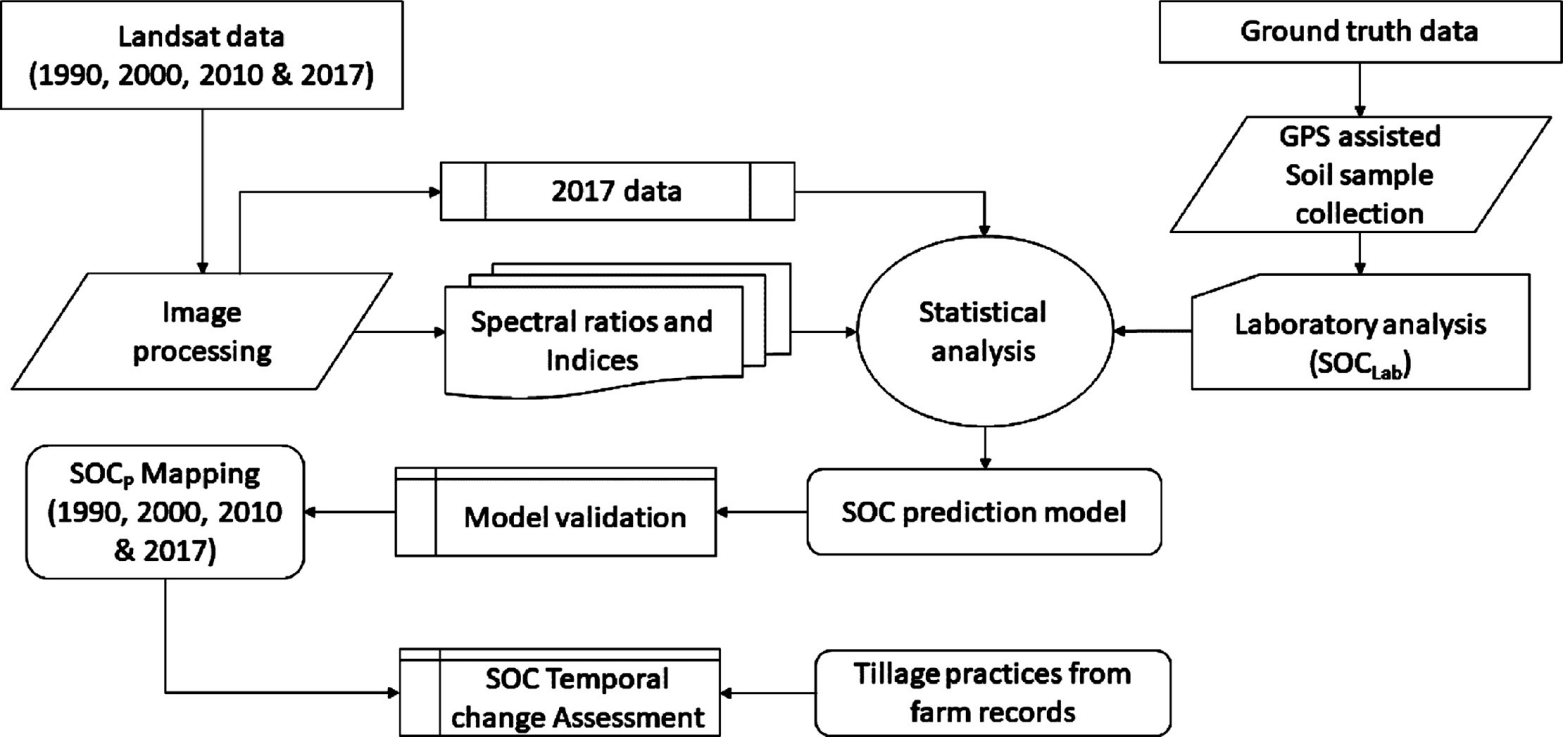
Procedure of soil organic carbon mapping.

The methodological flow chart involved in mapping the SOC and assessing its temporal changes associated with soil tillage practices over the period from 1990 to 2016 is presented in Fig 2. The laboratory determined SOC (SOC_Lab_), of the soil samples collected during a field campaign (March 27^th^, 2017), and the corresponding reflectance extracted from Landsat-8 (L8) satellite image of March 26^th^, 2017 were utilized for the development of SOC prediction models using the multiple linear regression analysis of the SPSS (Ver. 20) software program. The approach followed in this study included ground truth SOC data collection, satellite image analysis, development and validation of SOC prediction models/maps and assessment of the temporal changes in the SOC content of the experimental fields associated with the adopted soil tillage practices over the study period (1990–2016). Table 2 provides the inputs utilized for the development of SOC prediction models and the assessment of SOC temporal changes.

**Table 2:** Inputs used for the development of SOC prediction models.

	Parameter/Product	Source
1	SOC (g kg^-1^) content from soil samples	Laboratory Analysis
2	Landsat images (TM, ETM+, L8)	USGS

### Soil sampling and SOC determination

A field sampling was carried out on March 27^th^, 2017 and a total of 203 soil samples were randomly collected from the top soil layer (0 to 10 cm) of the six experimental fields (F1 to F6). A hand-held GPS receiver (Trimble GeoXH) was used for the identification of the pre-defined sampling locations (Fig 1). The sampling strategy was designed according to the stratified random sampling method [24]. The field elevation, slope and vegetation cover layers were considered in the stratification. The collected soil samples were first air-dried, gently crushed with a mortor and pestle, passed through a 2 mm sieve and stored in plastic containers. The SOC content of fine soil (Ø 2 mm) was then estimated, using the classic dichromate oxidation method of Walkley-Black [25], at the lab facility of the Preision Agriculture Research Chair (PARC), King Saud University, Saudi Arabia. The test was repeated three times for each sample and the resulting values were averaged out. The amount of ferrous ammonium sulphate (FAS) consumption during the titration was used to determine the SOC, as given in Eq 1.

$$SOC(\%)=N\times (B-S)\times 0.003\times \frac{100}{soil\;weight}$$ (1)

where, N is the normality of standard ferrous ammonium sulfate (0.5N), B and S are the amounts of ferrous ammonium sulphate utilized during the titration for blank and soil sample, respectively. Subsequently, the obtained SOC_Lab_ (%) values were transformed into unit values (g kg^-1^).

### Satellite data and image analysis

Cloud-free images of Landsat (TM, ETM+, L8-OLI) were downloaded from the USGS portal (https://earthexplorer.usgs.gov) and analyzed using the ENVI (Ver. 5.2) software program. The acquired Landsat images (details of which are provided in Table 3) were preprocessed for atmospheric corrections and radiometric calibration. Subsequently, the images were subset to the area of interest (the experimental farm). In case of the SLC-off image of the year 2010, three consequent images (multiscene SLC-off) were used to fill in the gap utilizing the Gap-Fill algorithm of the USGS Phase 2. The processed reflectance images were used for the generation of the predictor variables (i.e.NDVI and BSI) used for SOC mapping. Landsat images were co-registered and georeferenced to the Universal Transverse Mercator (UTM) map projection with the World Geodetic System 84 (WGS84) datum. Image to image rectification was applied for image correction. The Landsat-8 image acquired on March 26^th^, 2017 was considered as the reference (base/master) image. During the geometric correction; however, the total root mean square error (RMSE) of the rectification was maintained at values lower than 0.25 pixels. The linear contrast stretch function was applied to enhance the contrast and clarity of the image.

**Table 3:** Details of the sensors, the acquired images and the corresponding tillage practices.

Satellite/sensor	Path / row	Date of overpass	Tillage practice
Landsat (TM)	164/44	28 February 1990	Conventional Tillage
Landsat (ETM+)	165/44	19 March 2000	Conventional Tillage/ initiation of Conservation Tillage
Landsat (ETM+)	165/44	05 & 31 March 2010	Transition to Conservation Tillage
Landsat-8 (OLI)	165/44	23 March 2016 & 26 March 2017	Stabilized Conservation Tillage

### Vegetation indices and SOC mapping

The NDVI and BSI maps were generated utilizing the bandmath tool of the ENVI (ver.5.2) software program according to Eq 2 [26] and Eq 3 [27]. The study was mainly focused on the cultivated fields. The SOC was estimated using the empirical models of the generated BSI and NDVI layers. For the fields with crops at the time of satellite image, the NDVI was used as an input for SOC estimation. High BSI values indicated bare soil condition or fallow fields, where, moderate BSI values indicated a recently sown/harvested field that was partially covered with vegetation and crop residues.

NDVI=(NIR−Red)/(NIR+Red) (2)

$$BSI=\frac{\left[(SWIR+Red)-(NIR+Blue)\right]}{\left[(SWIR+Red)+(NIR+Blue)\right]}\times 100+100$$ (3)

where, the Blue, Red, Near Infra-Red (NIR) and Short Wave Infra-Red (SWIR) are the spectral reflectance values captured by respective Landsat bands.

### Calibration and cross-validation of SOC prediction models

The lab estimated SOC and the BSI and the NDVI, obtained from the Landat-8 image acquired on March 26^th^, 2017, were subjected to regression analysis for the development of the SOC prediction models. Before embarking on the regression analysis, the obtained dataset (204 samples) was randomly divided into two subsets of 142 samples (70% of the whole dataset) and 61 samples (30% of the whole dataset) for model development and cross-validation, respectively. The analysis of variance (ANOVA) statistics tool was used to test the strength of the developed models. The models that exhibited the strongest relationship between the SOC_Lab_ and the vegetation indices (The NDVI and the BSI) were subjected to further accuracy assessment using selected statistical performance indicators, including the R^2^, The P-value, the mean bias error (MBE) and the root mean square error (RMSE).

### Assessment of temporal changes in the SOC

Image substraction method [28] was utilized for the estimation of the temporal changes in SOC content of the selected agricultural fields during the periods 1990–2000, 2000–2010 and 2010–2016. For example, the potential SOC stock change map (SOC_1990-2010_) was obtained by subtracting the SOC_1990_ from the SOC_2000_ divided by the SOC_1990_ to determine the relative change (SOC_1990–2000_, %). Further, the magnitude of changes that could represent the variation in the SOC stock was addressed at the pixel level using the minimum detectable difference (MDD) approach [29, 30]. Subsequently, the results were classified into (a) positive change, (b) no-change and (c) negative change areas of SOC content.

## Results and discussion

### SOC prediction models

The laboratory determined SOC (SOC_Lab_) and the corresponding values of the vegetation indices (VIs), namely, the BSI and the NDVI extacted from the Landsat data were utilized for the development and cross-validation of SOC prediction models. Table 4 shows the descriptive statistics of the collected observations. Results showed high varibility in the SOC content across the experimental fields indicated by the SOC values ranging between 0.62 and 14.98 g kg^-1^ and the coefficient of variation (CV) values ranging between 39.57% and 58.43%. The variability in the NDVI and the BSI across the experimental fields was relatively low, except for the NDVI at low vegetation condition with a CV of 33.33%.

**Table 4:** Descriptive statistics of the lab determined SOC (g kg^-1^) and the generated VIs.

	Low Vegetation Condition			High Vegetation Condition		
	SOC_Lab_	NDVI	BSI	SOC_Lab_	NDVI	BSI
No. of Samples	98	98	98	105	105	105
Min	0.62	0.09	105.66	0.68	0.38	69.97
Max	14.98	0.36	126.31	24.25	0.57	96.45
Mean	7.53	0.18	118.22	15.92	0.51	78.25
SE	0.44	0.01	0.57	0.61	0.01	0.58
SD	4.40	0.06	5.68	6.30	0.05	5.98
CV, %	58.43	33.33	4.80	39.57	9.80	7.64

The collected SOC_Lab_ observations and the corresponding VIs (NDVI and BSI) generated from the Landsat-8 image of March 26^th^, 2017 were subjected to regression analysis. The obtained empirical models and the cross-validation results are presented in Tables 5 and 6.

**Table 5:** SOC-VIs regression models.

Model	Equation	Field Condition
**Model—1**	SOC_p_ = (-0.687 * BSI) + 88.497	Bare soil
**Model—2**	SOC_p_ = 72.911 + (16.035 * NDVI)–(0.578 * BSI)	Low Vegetation
**Model—3**	SOC_p_ = (124.145 * NDVI) - 47.782	High Vegetation

**Table 6:** SOC-VIs modeling and cross validation statistical results.

Parameter	Modeling Results			Cross-Validation Results		
	Model—1	Model—2	Model—3	Model—1	Model—2	Model—3
**R**	0.85	0.87	0.92	0.80	0.84	0.78
**R**^**2**^	0.73	0.76	0.85	0.64	0.70	0.61
**Adj. R**^**2**^	0.72	0.75	0.85	0.62	0.69	0.60
**P-Value**	0.0001	0.0001	0.0001	0.0001	0.0001	0.0001
**RMSE**				0.85	0.69	0.34
**MBE**				0.72	0.48	0.23

These results exhibited three empirical models, namely, model-1 representing the bare soil condition (NDVI ≤ 0), model-2 representing the low vegetation condition (0 ˂ NDVI ≤ 0.3) and model-3 representing the high vegetation condition (NDVI > 0.30). These models showed high significant relationships between the SOC and the VIs, indicated by the high R^2^ values of 0.73–0.85 and low P-value (≤ 0.0001). The cross-validation results of the developed models also confirmed the high significant correlation between the SOC and the VIs, with R^2^ values ranging between 0.61 and 0.70 and a P-value ≤ 0.0001. These significant linear relationships between the SOC and the VIs (NDVI & BSI) are in agreement with earlier studies [31, 32, 33, 34]. Overall, the statistical results indicated that these models could significantly predict the SOC content of agricultural fields from Landsat satellite data.

### Prediction of the SOC across the study period

**Fig 3:**
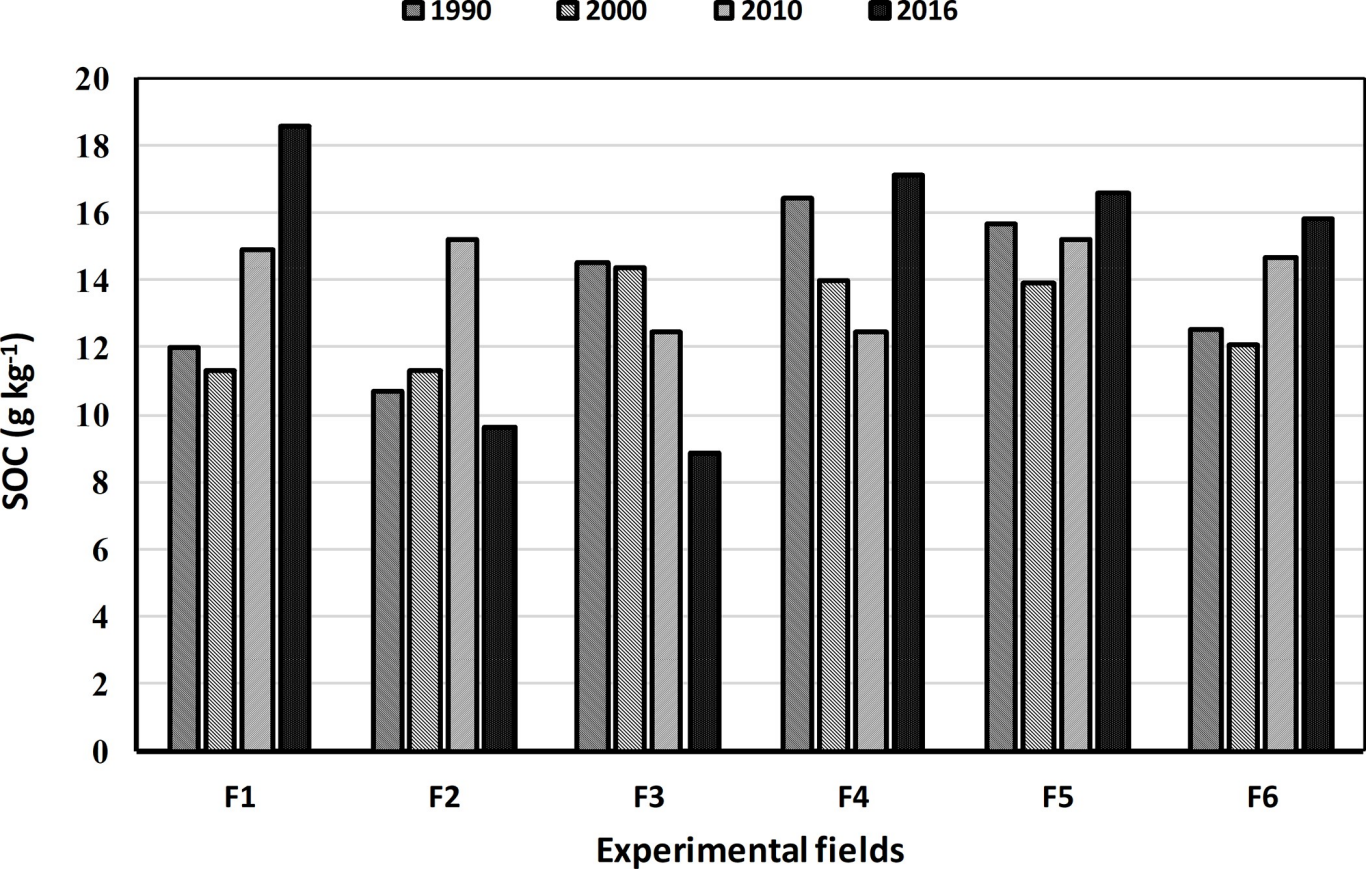
Mean values of the SOC content of the experimental fields during the study period.

The developed SOC carbon prediction models were utilized for maping SOC stocks of the experimental fields for the years 1990, 2000, 2010 and 2016. The descriptive statistics data (Table 7) indicated that the SOC content across the experimental fields ranged from 5.57 g kg^-1^ (F1) to 19.56 g kg^-1^ (F2) during the study period. The CV range of 0.36% to 27.89% indicated a moderate variability in the SOC content across the experimental fields. As illustrated in Fig 3, the mean values of the SOC content of the experimental fields showed a positive improvement by the end of the study period (2016), in the fields F1, F4, F5 and F6. The temporal changes of the SOC content is thoroughly discussed in the next section.

**Table 7:** Descriptive statistics of the SOC (g kg^-1^) content of the experimental fields.

Field No.	Year	Min.	Max.	Mean	SD	SE	CV, %
**F1**	**1990**	7.26	14.92	12.02	2.21	0.37	4.87
	**2000**	8.93	12.54	11.29	0.86	0.15	0.74
	**2010**	5.57	18.14	14.91	3.52	0.59	12.37
	**2016**	15.05	19.08	18.58	0.68	0.11	0.46
**F2**	**1990**	5.87	19.56	10.69	5.28	0.89	27.89
	**2000**	8.96	12.53	11.31	0.90	0.15	0.81
	**2010**	7.20	17.63	15.16	2.19	0.37	4.81
	**2016**	8.24	12.90	9.59	1.57	0.27	2.46
**F3**	**1990**	10.91	18.21	14.48	1.67	0.29	2.79
	**2000**	11.68	15.23	14.35	0.77	0.13	0.59
	**2010**	9.05	18.86	12.45	2.44	0.43	5.97
	**2016**	8.13	10.58	8.88	0.68	0.12	0.46
**F4**	**1990**	14.79	18.15	16.45	0.68	0.12	0.46
	**2000**	12.31	15.11	14.01	0.71	0.12	0.51
	**2010**	9.15	15.47	12.48	1.32	0.23	1.75
	**2016**	14.35	18.04	17.12	0.75	0.13	0.56
**F5**	**1990**	11.23	17.83	15.64	1.48	0.26	2.19
	**2000**	11.13	14.71	13.87	0.74	0.13	0.55
	**2010**	10.58	17.87	15.16	1.87	0.33	3.49
	**2016**	10.66	17.24	16.57	1.09	0.19	1.18
**F6**	**1990**	8.17	19.43	12.53	4.67	0.81	21.84
	**2000**	10.59	13.28	12.08	0.60	0.10	0.36
	**2010**	11.89	16.16	14.63	1.14	0.20	1.31
	**2016**	8.12	18.25	15.83	2.90	0.50	8.38

### Tillage practices induced temporal changes in the SOC content

Temporal changes in the SOC content of the experimental fields, during the study period are presented in Table 7 and Fig 3. The change in SOC values across the study period was clearly matched with the adopted tillage practices. During the period 1990–2000, where conventinal tillage was practiced, a decrease of 0.90 to 15% in the SOC content was observed in almost all the six experimental fields, except in case of F2 where an increase of about 6% in the SOC content was recorded. Altough, the application of conservation tillage over the period of ten years (2000–2010) increased the amount of the SOC by 9 to 34% in four (F1, F2, F5 and F6) of the six experimental fields, a decrease of 13% and 11% in was recorded in fields F3 and F4, respectively. While, in the period of stabilized conservation tillage application (2010–2016), an increase in the SOC content of 8 to 37% was recorded in four out of the studied six fields (F1, F4, F5 and F6). The reduction in the SOC content in the other two fields (F1 and F3), however, was attributed to the discontinuation of agricultural practices in these fields for the last four years. In general, the adoption of conservation tillage showed overall increase of 4–55% in the SOC content at the end of the study period (2016) compared to that at the beginning of the study period (1990), in about 67% of the studied fields (F1, F4, F5 and F6).

**Fig 4:**
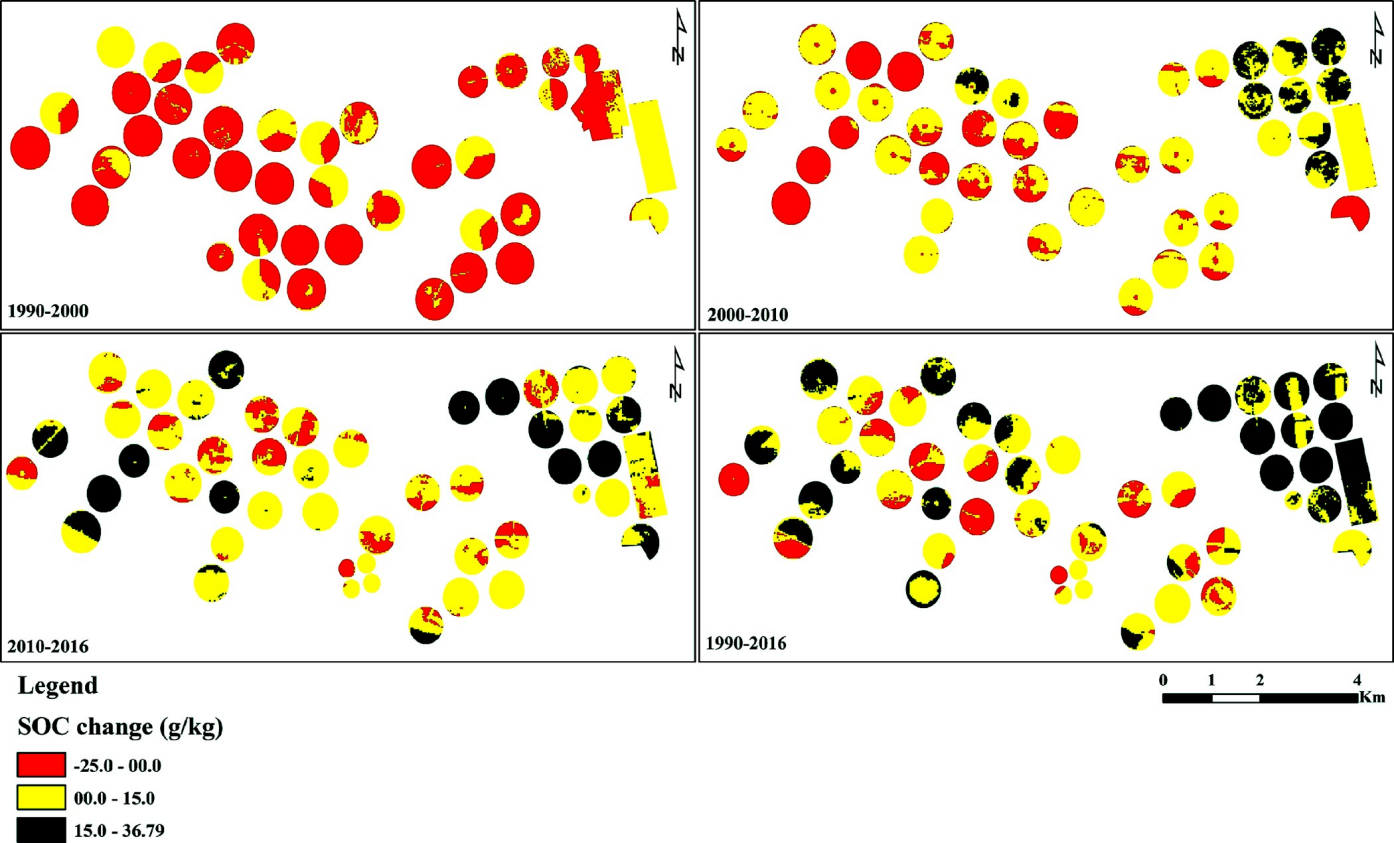
Changes in the SOC content of the experimental fields during the study period.

**Fig 5:**
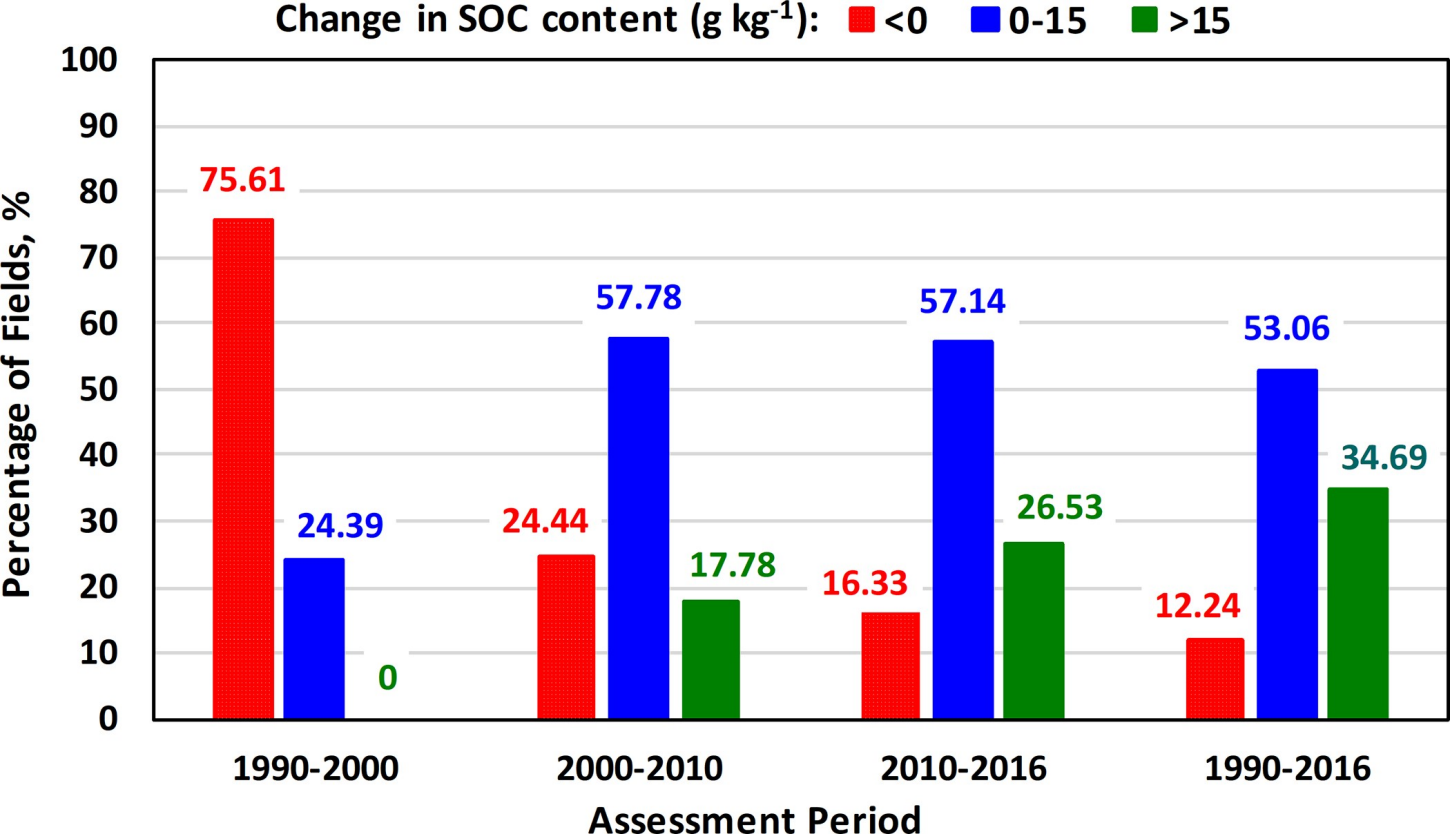
Temporal changes of SOC content across the the experimental farm.

To study the overall impact of soil tillage on the SOC content in all agricultural fields of the study farm (49 fields), the trend of the amount of the SOC was categorized into three groups, namely, high positive change (≥15 g kg^-1^), low positive change (0–15 g kg^-1^) and negative change (<0 g kg^-1^) as indictated in Figs 4 and 5. During the period 1990–2000, where conventional tillage was the dominant tillage practice, about 76% of the fields showed SOC reduction of up to 24 g kg^-1^. While, 76% and 84% of the agricultral fields in the experimental farm showed an increase in the SOC content during the periods 2000–2010 and 2010–2016, respectively. In general, the positive change, of up to 37 g kg^-1^, in the SOC content of 88% of the agricultural fields of the experimental farm during the period from 2000 to 2016, was attributed to the adoption of conversion of tillage practices.

Results of this study were in agreement with the findings of previous studies, which reported a significant improvement in the SOC content as a result of conservation tillage practices. Dikgwatlhe et al. [6] reported that the carbon concentration in the top soil layer (0–5 cm depth) under no-till was higher by 8–18% compared to that under plow tillage system. Results of the study conducted by Haddaway et al. [35] also revealed that the C stock in the upper soil layer (0–30 cm) increased by an average amount of 4.6 Mg ha^-1^ under no-till compared to high tillage intensity over a period of more than 10 years. The same was reported by Yeboah et al. [36], where the no-till system was found to increase the SOC in the soil depths of 0–5 cm, 5–10 cm and 10–30 cm by 19%, 25% and 7%, respectively, compared to the conventional tillage system. Furthermore, the average SOC stock in soil depths of 0–10 cm and 0–20 cm, reported by Hernanz et al. [37], was significantly higher under zero tillage (ranging from 15.6 to 26.6 Mg ha^-1^) than under conventional tillage (ranging from 11.0 to 22.2 Mg ha^-1^) and minimum tillage (ranging from 11.8 to 22.7 Mg ha^-1^). Similar results were reported by Choudhury et al. [38], where zero tillage was found to improve SOC sequestration by 33.6% compared to conventional tillage after five years of continuous rice–wheat cultivation in sandy loam soil.

For a reliable application of these results, it is very important to consider the limitations on applying the SOC prediction models, generated from Landsat-8 (OLI) for the year 2017, to other to other satellite data (TM, ETM + and OLI) acquired for the years 1990, 2000 and 2010. For example, multi-temporal satellite data, acquired by different sensors under different conditions, may have reflectance inconsistencies due to various factors including atmospheric changes, sun-surface-sensor geometry, sensor degradation and calibration, variation in spectral band pass and spatial resolution etc. [39]. In this regard, a study on the technical variation between the L8 and ETM+ datasets, conducted by Mishra et al. [40] and Roy et al. [39], concluded that the L8 (OLI) performs better (±2.5%) than ETM+ in both radiance and reflectance space. They attributed that to higher uncertainties in the NIR and SWIR-1 bands (~1%) and other bands (<0.5%).

## Conclusions

A field study was conducted to employ GIS techniques to study the effect of tillage practices on the long-term (1990–2016) changes in the SOC content of the topsoil layers (0–10 cm) of agricultural fields in the central region of Saudi Arabia. The specific conclusions of this study are as follows:

- Landsat datasets were beneficial for SOC mapping. The developed SOC prediction models showed high accuracy indicted by the R^2^ values of 0.73–0.85, the RMSE values of 0.34–0.85 g kg^-1^ and the P-values of ≤ 0.0001. The cross-validation results (R^2^ of 0.61–0.70, RMSE value of 0.34–0.85 g kg^-1^ and P-values of less than 0.0001) confirmed the high accuracy of the developed SOC prediction models.
- Results of the study proved that the change in the SOC content was clearly associated with soil tillage practices. About 76% of the agricultural fields in the experimental farm showed a decrease of up to 24 g kg^-1^ in the SOC content over the period from 1990 to 2000 as a result of conventional tillage practices.
- An average increase of up to 37 g kg^-1^ in the SOC content was observed in 88% of the studied fields at the end of the study period (2016), where conservation tillage was a continous and consistent practice in the experimental farm

## Supporting information

S1 File SOC_Stat_6 Fields. (XLSX)

S2 File SOC_Modelling-6 Fields. (XLSX)

S3 File SOC_Change_All Fields. (XLSX)

S4 File Protocol- soil carbon mapping. (PDF)
