# Supplementary material for: Employment of GIS techniques to assess the long-term impact of tillage on the soil organic carbon of agricultural fields under hyper-arid conditions
Source: PLoS One. 2019 Feb 19;14(2):e0212521. doi: 10.1371/journal.pone.0212521 (PMC6380592; doi:10.1371/journal.pone.0212521)
Supplement: S4 File — (PDF) [file pone.0212521.s004.pdf]

Soil Organic Carbon Mapping

A) Soil sampling

- *Landsat-8 image & ASTER DEM download.*
- *Generation of slope, elevation and vegetation cover layers.*
- *Stratified random sampling (sampling points).*
- *GPS assisted soil sampling (point locations)*

B) Soil Organic Carbon (SOC_{Lab}) Analysis

1. Sample Preparation:

- Air-drying of the collected soil samples.
- Sieve the samples through a 2 mm sieve.
- Store the samples in a labelled plastic boxes/bottles (stick the labels on the box and lid).
- Crush 25 g of fine sample in a mortar and pass it through a 0.25 mm sieve.

2. Method used for Soil Organic Carbon Analysis

- The *Walkley-Black* procedure
- Wet combustion of the organic matter with a mixture of potassium dichromate and sulphuric acid at 125°C.
- The residual.

3. Apparatus

- Burette.
- Safety pipette (10 ml).
- Illuminated magnetic stirrer.
- Measuring cylinder (25 ml).

4. Reagents:

- *Potassium dichromate (standard solution, 0.1667 M):* dissolve 49.04g $K_2Cr_2O_7$ A.R. (dried at 105°C) in water in a 1.0 liter volumetric flask.
- *Concentrated sulphuric acid (96%)*
- *Concentrated phosphoric acid (85%)*
- *Barium diphenylamine sulphate, 0.16% (indicator):* dissolve 1.6g barium diphenylamine sulphate in 1 liter water.
- *Ferrous sulphate solution, 1 M (approx.):* dissolve 278g FeSO4.7H2O in ca. 750 ml water and add 15 ml conc. H_2SO_4. Transfer to a 1.0 liter volumetric flask.

5. Procedure

- Preparation of fine samples passing a 0.25 mm sieve.
- Weigh 1.0 g of soil into a 500 ml wide-mouth Erlenmeyer flask.
- Add 10 ml potassium dichromate solution.
- Take another two Erlenmeyer flasks without soil and add 10 ml potassium dichromate solution to determine the molarity of ferrous sulphate solution (as a blanks).

- Carefully add 20 ml sulphuric acid with a measuring cylinder (for all flasks), swirl the flask and allow to stand on a pad for 30 min.
- Add 250 ml water and 10 ml phosphoric acid and allow to cool.
- Add 1.0 ml indicator solution and titrate with ferrous sulphate solution while the mixture is being stirred. Near the end point, where the brown colour becomes purple or violet-blue, slow down the titration process. At the end point, the colour will change sharply to green.

6. Calculation:

- *The carbon content of the soil is obtained by:*

$$\%C = M\ x\ \frac{V1 - V2}{s}\ x\ 3.9\ x\ mcf$$

Where
M = molarity of ferrous sulphate solution (from blank titration)
V1 = ml ferrous sulphate solution required for blank
V2 = ml ferrous sulphate solution required for sample
S = weight of air-dry sample in gram
0.39 = 3 x 10^{-3} x 100% x 1.3 (3 = equivalent weight of carbon)
Mcf = moisture correction factor

C) Data preparation

- *SOC_{Lab} data coding to GPS data.*
- *Rasterization of SOC_{Lab}*
- *Data splitting (a) Test subset; (b) Validation subset.*
- *Landsat (TM, ETM+ & OLI) data download and image analysis using ENVI.*
- *NDVI and BSI layers generation.*

D) Modelling and cross-validation

- *Multiple linear regression analysis (y-axis: SOC_{Lab}), (x-axis: BSI and NDVI from Landsat-8(OLI).*
- *Cross-validation.*
- *Model prediction and SOC_P mapping.*

E) SOC change analysis (based on the tillage practices)

- *Landsat-8 derived SOC prediction models integrated with TM, ETM+ data after the correction with empirical equations (Landsat OLI Vs. TM and ETM+ reflectance correlation models).*
